# Exertional Desaturation Has Higher Mortality Than Non-Desaturation in COPD

**DOI:** 10.3390/medicina57101110

**Published:** 2021-10-15

**Authors:** Shih-Feng Liu, Chien-Hung Chin, Ching-Wang Tseng, Yung-Che Chen, Ho-Chang Kuo

**Affiliations:** 1 Division of Pulmonary and Critical Care Medicine, Department of Internal Medicine, Kaohsiung Chang Gung Memorial Hospital, Kaohsiung 833, Taiwan; chestman57112@yahoo.com.tw (C.-H.C.); yungchechen@yahoo.com.tw (Y.-C.C.); 2 Department of Respiratory Therapy, Kaohsiung Chang Gung Memorial Hospital, Kaohsiung 833, Taiwan; wanda2640@cgmh.org.tw (C.-W.T.); erickuo48@yahoo.com.tw (H.-C.K.); 3 Medical Department, College of Medicine, Chang Gung University, Taoyuan 333, Taiwan; 4 School of Medicine, Chung Shan Medical University, Taichung 40201, Taiwan; 5 Department of Pediatrics, Kaohsiung Chang Gung Memorial Hospital, Kaohsiung 833, Taiwan

**Keywords:** exertional desaturation, COPD, DLCO, mortality, 6MWT

## Abstract

*Background and objectives:* Exertional desaturation (ED) is often overlooked in chronic obstructive pulmonary disease (COPD). We aim to investigate the impact of ED on mortality and the predictors of ED in COPD. *Materials and* *methods:* A cohort of COPD patients with clinically stable, widely ranging severities were enrolled. ED is defined as oxyhemoglobin saturation by pulse oximetry (SpO2) < 90% or a drop of ΔSpO2 ≥ 4% during a six-minute walk test (6MWT). Cox regression analysis is used to estimate the hazard ratio (HR) for three-year mortality. *Results:* A total of 113 patients were studied, including ED (*N* = 34) and non-ED (*N* = 79) groups. FVC (% of predicted value), FEV1/FVC (%), FEV1 (% of predicted value), DLCO (%), maximal inspiratory pressure, SpO2 during the 6MWT, GOLD stage, and COPD severity were significantly different between the ED and non-ED groups in univariate analysis. Low minimal SpO2 (*p* < 0.001) and high maximal heart rate (*p* = 0.04) during the 6MWT were significantly related to ED in multivariate analysis. After adjusting for age, gender, body mass index, 6MWD, FEV1, mMRC, GOLD staging, exacerbation, hs-CRP, and fibrinogen, the mortality rate of the ED group was higher than that of the non-ED group (*p* = 0.012; HR = 4.12; 95% CI 1.37–12.39). For deaths, the average survival time of ED was shorter than that of the non-ED group (856.4 days vs. 933.8 days, *p* = 0.033). *Conclusions*: ED has higher mortality than non-ED in COPD. COPD should be assessed for ED, especially in patients with low minimal SpO2 and high maximal HR during the 6MWT.

## 1. Introduction

Exertional desaturation (ED) in patients with chronic obstructive pulmonary disease (COPD) is a common clinical condition [1,2]. However, the prevalence of ED is uncertain. There are many reasons for this condition due to the different definitions of ED, degree of exercise, different forms of exercise examination, COPD heterogeneity, and differently selected study population [2]. ED is easily overlooked by clinicians, especially in COPD patients with normal blood oxygen saturation while at rest. Exercise may actually improve the gas exchange in subjects with mild COPD, which is mainly due to a more even distribution of ventilation, leading to an improvement in the ventilation (V)/perfusion (Q) ratio [3]. However, ED is frequently encountered in more severe disease. The causes for ED in COPD patients are multifactorial and include V-Q mismatches, poor diffusing capacity of the lungs for carbon monoxide (DLCO), shunting with reduced oxygen content of mixed venous blood, and even nonuniform standardized exercise protocols. Some studies have tried to identify the predictors for ED in COPD, but the results have been mixed [1,4,5,6,7,8,9,10,11,12,13,14]. Resting oxygen saturation and lung function studies may not be able to reliably predict which patients with COPD will develop ED.

Chronic hypoxia in COPD is often accompanied by poor exercise tolerance and quality of life and increased cardiovascular morbidities and cardiovascular all-cause mortality [15]. Evidence also demonstrates that ED is usually associated with poor clinical outcomes [14,15,16]. Although the benefit of ambulatory oxygen therapy for COPD patients with mild to moderate ED is controversial [17,18,19,20,21], the six-minute walk test (6MWT) provides clinicians with an indication of the presence of ED and a treatment guideline for selected patients with COPD and severe hypoxemia on exertion.

This study was conducted using a cohort of stable COPD patients, tracked over three years. We conducted an analysis to assess the effect of ED on the mortality of patients with stable COPD and to determine the clinical variables related to ED.

## 2. Materials and Methods

### 2.1. Study Design

This observation study was conducted over three years. The purpose of the study was to evaluate the effect of ED on the mortality of a group of patients with stable COPD and to identify clinical variables as predictors of ED. ED was defined as a fall in oxyhemoglobin saturation by pulse oximetry (SpO2) to <90% or a decrease of ΔSpO2 ≥ 4% during the 6MWT [13]. We divided the participants into two groups: the ED group and the non-ED group. Variables included age, gender, current tobacco use, pack-years, maximum inspiratory pressure, maximum expiratory pressure, severity of COPD, modified Medical Research Council dyspnea scale (mMRC), body mass index (BMI), DLCO, 6MWD, serum high-sensitivity CRP (hs-CRP), and fibrinogen concentration, as well as body mass index, airflow obstruction, dyspnea, and exercise capacity (BODE) score.

### 2.2. Study Participants

A total of 113 patients with various COPD severities were selected continuously from the Pulmonary Clinic in Chang Gung Memorial Hospital—Kaohsiung Medical Center. These patients underwent lung function tests and a 6MWT according to recommendations published by the American Thoracic Society. Each COPD patient was over 40 years of age, with a high rate of smoking and a minimum of 10 years of smoking history. The diagnosis of COPD was based by GOLD guideline. For avoiding confounding factors related to ED, we excluded some diseases using radiology and chart review. In selected patients, other causes of airway obstruction, such as tuberculosis, bronchial asthma, bronchiectasis, and heart failure, were ruled out by viewing chest X-rays and medical documents. We also reviewed medical records to exclude patients diagnosed with cardiovascular disease, pulmonary hypertension, peripheral vascular disease, cerebrovascular disease, collagen vascular or interstitial lung disease. Patients experiencing any exacerbation of COPD or hospitalization within 6 weeks prior this study were excluded. The follow-up duration of these patients was from 730 days to 1200 days, individually. During follow-up, mortality for any reason was used as an analysis variable.

### 2.3. Ethical Approval and Patient Informed Consent

The study was approved by the Chang Gung Memorial Hospital Review Board (IRB # 94-319). Written informed consent from each participant was obtained. All methods were carried out in accordance with relevant guidelines and regulations.

### 2.4. Statistics

Continuous variables are expressed as mean ± standard deviation, and categorical variables are expressed as absolute values and percentages. Univariate and multivariate analyses between the ED and non-ED groups were performed using Student’s t and linear regression tests. In the multivariate analysis, we reanalyzed the variables with a *p*-value < 0.1 in the univariate analysis. The cumulative survival rate was estimated by the Kaplan–Meier method using the log-rank test. Cox regression analysis was used to estimate the hazard ratio of mortality (HR). Two-tailed *p* < 0.05 was considered statistically significant. The SPSS program (version 22.0; SPSS Inc., Chicago, IL, USA) was used for statistical analysis.

## 3. Results

### 3.1. The Characteristics of the Participants

We include a total of 113 patients with stable COPD (ED group (*N* = 34, 30.1%) and non-ED group (*N* = 79, 69.9%). Table 1 shows the characteristics of the study participants. The variables of the characteristics include age, gender, smoking history, lung function, BMI, GOLD stage, MMRC scale, and exercise capacity (6MWD).

### 3.2. Risk Factors with ED

Table 2 shows the characteristics of the COPD patients in the ED and non-ED groups during the 6MWT. FVC (% of predicted value), FEV1/FVC (%), FEV1 (% of predicted value), DLCO (%), maximal inspiratory pressure (MIP), SpO2 during the 6MWT, GOLD stage, and COPD severity were significantly different between the ED and non-ED groups in the univariate analysis. Minimal SpO2 (84.2% vs. 93.0%; *p* < 0.001; 95% CI: −0.08–−0.05), and maximal HR (136.8 vs. 120.9/min; *p* = 0.04; 95% CI: 0.00–0.07) during the 6MWT were significantly associated with ED by multivariate analysis.

### 3.3. ED Is a Predictor of Mortality in Patients with Stable COPD

**Figure 1:**
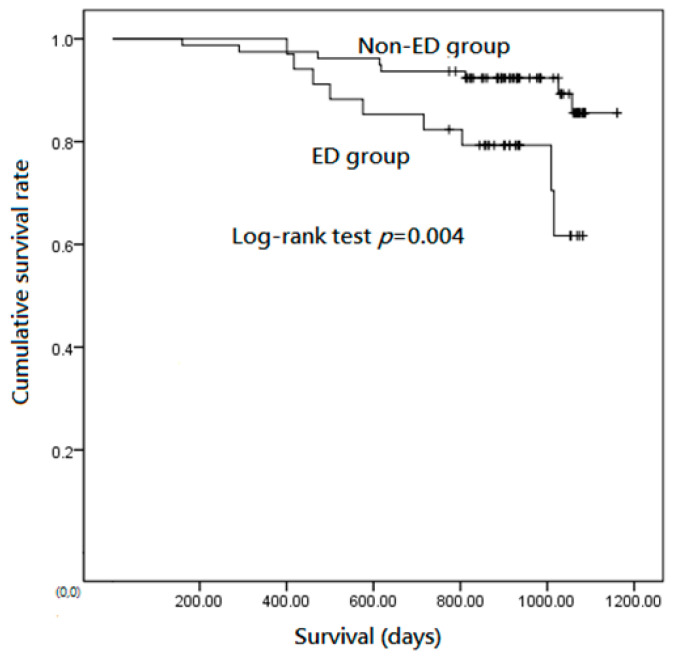
Kaplan–Meier analysis showed the cumulative survival rates of the exertional desaturation (ED) group were significantly lower than that of the non-ED group.

There was a significant difference in the average survival time between the ED group and the non-ED group (856.4 days vs. 933.8 days, *p* = 0.033). Kaplan–Meier analysis showed that the cumulative survival rate of the ED group was significantly lower than that of the non-ED group (Figure 1). Table 3 showed the results of univariate analysis and multivariate analysis of mortality in patients with stable COPD. Age (*p* = 0.028; Exp(B) = 1.08; 95% CI: 1.01–1.17), MMRC scale (*p* = 0.027; Exp(B) = 2.01; 95% CI: 1.08–3.71), serum hs-CRP < 3mg/L (*p* = 0.017; Exp(B) = 0.22; 95% CI: 0.06–0.76), and ED (*p* = 0.012; Exp(B) = 4.12; 95% CI: 1.37–12.39) were significantly associated with mortality in the multivariate analysis.

## 4. Discussion

Our current study demonstrated that the incidence of ED during the 6MWT was approximately 30% in patients with COPD of various severities, and the ED group had higher mortality than the non-ED group in COPD. ED was significantly associated with low minimal SpO2 and high maximal HR during the 6MWT.

COPD is a leading cause of mortality worldwide. Current data indicate that the severity of this disease is not only graded by airflow limitation according to the FEV1, but is also evaluated by BMI, degree of dyspnea, 6MWD, and exacerbation frequencies in patients with COPD [22,23].

The 6MWT has been found to be more sensitive than maximum incremental cycle testing for the detection of oxygen desaturation; it has become the preferred test for the evaluation of oxygen requirements in COPD [24,25,26]. ED prevalence of 29.1% was noted in moderate to very severe COPD patients in the study by Stolz et al., and ED was associated with a greater deterioration of the health-related quality of life, higher severe exacerbation, and higher annual death rates [1]. Barbera et al. noted the significant correlations between the severity of the pathologic findings and both the degree of hypoxemia and the extent of V/Q mismatching at rest. However, the severity of emphysema was correlated with PaO2 during exercise [4]. Dynamic hyperinflation is closely related to ED and may be the cause of ED; meanwhile, the results of routine pulmonary function tests do not correlate well with ED in stable COPD [9].

DLCO is an excellent index of the extent of disseminated emphysema in smokers with airway obstruction. Low DLCO is associated with a lower average lung CT density and an anatomic emphysema [27,28]. These findings, consistent with our current findings, show that low DLCO is associated with ED. Low DLCO is a predictor of decreased oxygen saturation during exercise, and therefore the DLCO test may be used as a screening test for ED in patients with COPD [29,30]. Additionally, a combination of DLCO and BODE score greater than seven was recommended for the evaluation of desaturation during daily activities [8]. However, DLCO showed no significant relation with ED in by multivariate analysis in our study.

Even if there is no obvious resting hypoxemia, the daily activities of patients with moderate to severe COPD, such as walking, washing, and eating, are also associated with a transient decrease in oxygen saturation [31]. The possibility of exacerbation increases 50%, lung function is lost faster, and the mortality rate is doubled in COPD with ED compared to that without ED [32]. Waatevik et al. also found that ED was more likely to have fat-free weight loss during follow-up [32]. Therefore, SpO2 and pulse should be recorded before the test, throughout the test, and during the recovery period [33]. Our study also showed that COPD patients with low minimal SpO2 and high maximal HR during the 6MWT should be assessed for ED. SpO2 during a 6MWT identifies a COPD phenotype with an increased risk of morbidity and mortality [34]. The level of minimal SpO2 during a 6MWT related to the COPD outcome is worthy of further study.

There are some limitations to this study. First, this study was a post hoc analysis of our original study [35]. Although prospective data were used for the purpose of the original study, which investigated the variables related to mortality in patients with stable COPD, all data were complete, including 6MWT examinations (the degree of desaturation and heart rate, respiratory rate, and Borg scale changes during 6MWT), lung function, BMI, MMRC, hsCRP, fibrinogen, and other variables at follow-up for mortality. No additional data were collected retrospectively. Second, although we enrolled patients with stable COPD, they were predominantly chosen on the basis of their medical history and medical records to rule out cardiovascular disease. We did not actually use relevant examinations, such as a cardiac echocardiogram to evaluate cardiac function, to exclude possible comorbidities. These comorbidities might be important factors leading to ED during the 6MWT. Third, high-resolution computed tomography was not performed in this study. Factors affecting DLCO may be multifactorial. We only found no evidence of co-morbidity related to DLCO from the history and medical records, and we assumed that the decrease of DLCO was caused by COPD in this study.

## 5. Conclusions

ED leads to higher mortality than non-ED in COPD. COPD should be assessed for ED, especially in patients with low minimal SpO2, and high maximal HR during the 6MWT.

## Figures and Tables

**Table 1:** Basic clinical characteristics of 113 patients with stable chronic obstructive pulmonary disease.

Characteristic	Mean ± SD
Age (y)	69.5 ± 10.3
Male (%)	110/113 (97.3)
Smoking history (pack-y)	58.0 ± 31.8
Current smoking status (%)	35/113 (31.0)
Body mass index (BMI)	23.5 ± 3.7
FVC (% of predicted value)	71.7 ± 19.3
FEV1/FVC (%)	53.9 ± 11.5
FEV1 (% of predicted value)	53.1 ± 21.5
DLCO (%)	72.3 ± 24.2
Old GOLD stage	
I/II/III/IV	17//35/50/11
2012 GOLD	
Group A/B/C/D	31/22//10/50
mMRC dyspnea scale ^‡^	
Scale 0/1/2/3/4	17/25/29/30/31
6MWD (m)	402.4 ± 111.1

Abbreviations: FVC: forced vital capacity; FEV_1:_ forced expiratory volume in 1 s; COPD: chronic obstructive pulmonary disease; mMRC: the modified medical research council; 6MWD: 6 min walk distance; DLCO: diffusing capacity of the lungs for carbon monoxide. ^‡^ Scores on the modified Medical Research Council (mMRC) dyspnea scale range from 0 to 4, with a score of 4 indicating that the patient is too breathless to leave the house or becomes breathless when dressing or undressing. The body-mass index is the weight in kilograms divided by the square of the height in meters.

**Table 2:** Comparison of the characteristics of the ED and non-ED groups by univariate (*p*-value) and multivariate (*p* *-value) analyses.

	ED Group (*N* = 34)	Non-ED Group (*N* = 79)	*p* (95% CI)	*p* * (95% CI)
Age (Mean ± SD)	68.3 ± 9.7	70.1 ± 10.6	0.41	
Male (%)	97 ± 17	97 ± 15	0.90	
Smoking history(n)(%)	34(100)	79(100)	-	
Pack-yrs (Mean ± SD)	58.5 ± 33.7	57.7 ± 31.1	0.52	
Current smoking (n)(%)	10(29.5)	26(33)	0.91	
Body mass index (BMI)	23.2 ± 3.9	23.7 ± 3.6	0.79	
FVC (% of predicted value)	65.7 ± 19.3	74.3 ± 18.9	0.03 −16.0–0.3	0.80 −0.001–0.01
FEV1/FVC (%)	48.7 ± 11.1	56.1 ± 11.1	0.02 −16.2–−0.2	0.70 −0.19–0.13
FEV1 (% of predicted value)	43.7 ± 17.8	57.2 ± 21.7	0.02 −23.4–−6.1	0.9 −0.14–0.02
DLCO (%)	58.0 ± 17.0	78.4 ± 24.4	<0.001 −31.6–−12.6	0.5 −0.004–0.002
6MWD (m)	391.8 ± 109.9	411.6 ± 104.6	0.37	
MIP	75.3 ± 22.9	87.8 ± 31.8	0.04 −26.6–−1.9	0.76 −0.02–0.002
MEP	97.7 ± 31.6	99.2 ± 34.8	0.82	
SpO2 (initial) *	96.1 ± 1.9	96.6 ± 1.3	0.15	
SpO2 (end) ^#^	95.1 ± 4.2	96.2 ± 1.5	0.06 −2.2–0.03	0.5 −0.02–0.03
Minimal SpO2	84.2 ±5.4	93.0 ±1.7	<0.001 −10.6–−7.8	<0.001 −0.08–−0.05
HR (initial) *	82.9 ± 16.0	80.2 ± 15.1	0.4	
HR (end) ^#^	100.1 ± 17.2	94.9 ± 16.0	0.1 −1.3–12.3	
Maximal HR	136.8 ±21.5	120.9 ±19.1	<0.001 9.4–26.0	0.04 0.00–0.07
RR (initial) *	14.9 ± 3.5	14.7 ± 3.2	0.78	
RR (end) ^#^	18.7 ± 5.1	17.0 ± 4.1	0.06 0.4–3.8	0.65 −0.02–0.01
Borg scale (initial) ^*^	2.0 ± 1.0	2.1 ±1.1	0.78	
Borg scale (end) ^#^	2.9 ±1.2	2.8 ± 1.4	0.60	
ΔBorg scale	0.94 ± 0.2	0.73 ± 0.1	0.32	
Hs-CRP	3.8 ± 0.7	3.1 ± 0.4	0.37	
fibrinogen	319.3 ± 84.5	303.6 ± 76.0	0.34	
2012 GOLDGroup A/B/C/D	4/5/4/21	27/17/6/29	0.003 0.4–1.4	0.78 −0.09–0.11
FEV1 severity	2/7/16/9	15/28/34/2	<0.001 0.4–1.0	0.83 −2.35–0.19
MMRC	1/7/10/11/5	15/18/19/19/8	0.04 0.2–1.2	0.92 −0.07–0.07
Acute exacerbation	0.32 ± 0.08	0.23 ± 0.05	0.29	

Abbreviations: ED: exertional desaturation; COPD: chronic obstructive pulmonary disease; FEV_1:_ forced expiratory volume in 1 s; FVC: forced vital capacity; HR: heart rate; hsCRP: high-sensitivity C-reactive protein; MMRC: the modified medical research council; RR: respiratory rate; SaO2: oxygen saturation; 6MWD: 6 min walk distance. (initial) * means at the beginning of 6MWT; (end) ^#^ means at the end of 6MWT.

**Table 3:** Univariate analysis and multivariate analysis of mortality in patients with stable COPD by cox regression mode.

	*p* ^	Exp(B) ^	95% CI ^	p *	Exp(B) *	95% CI *
Age	0.023	1.06	1.01–1.12	0.028	1.08	1.01–1.17
Gender	0.38	0.05	0.00–60694	0.99	0	0
BMI	0.13	0.89	0.78–1.03	0.79	0.98	0.84–1.15
6MWD	0.01	0.995	0.991–0.995	0.82	1.001	0.99–1.01
FEV1(%)	0.55	0.99	0.99–1.02	0.88	1.004	0.95–1.06
MMRC scale	0.008	1.75	1.16–2.66	0.027	2.01	1.08–3.71
GOLD stage	0.32	1.22	0.82–1.84	0.82	1.001	0.995–1.01
AE	0.81	0.93	0.31–2.84	0.99	1.01	0.24–4.28
ED	0.007	3.68	0.11–0.70	0.012	4.12	1.37–12.39
hS-CRP < 3 mg/L	0.013	0.22	0.60–0.82	0.017	0.22	0.06–0.76

Abbreviations: COPD: chronic obstructive pulmonary disease; ED: exertional desaturation; FEV1: forced expiratory volume in 1 s; HR: heart rate; hsCRP: high-sensitivity C-reactive protein; MMRC: the modified medical research council; 6MWD: 6 min walk distance; ^: Univariate analysis *: Multivariate analysis.

## Data Availability

Our data can be obtained from the corresponding author: S.-F.L.

## Abbreviations

ED	exertional desaturation
COPD	chronic obstructive pulmonary disease
HRs	hazard ratios
DLCO	diffusing capacity of the lungs for carbon monoxide
6MWT	6 minute walking test
hsCRP	serum high-sensitivity C-reactive protein
V	ventilation
Q	perfusion ratio
MMRC	Modified Medical Research Council dyspnea scale
BMI	body mass index
BODE	body mass index, airflow obstruction, dyspnea, and exercise capacity
FVC	forced vital capacity
FEV1	forced expiratory volume in 1 s
RR	respiratory rate;
SpO2	oxyhemoglobin saturation by pulse oximetry.

## References

[1] Stolz D., Boersma W., Blasi F., Louis R., Milenkovic B., Kostikas K., Aerts J.G., Rohde G., Lacoma A., Rakic J. (2014). Exertional hypoxemia in stable COPD is common and predicted by circulating proadrenomedullin. Chest.

[2] Panos R.J., Eschenbacher W. (2009). Exertional desaturation in patients with chronic obstructive pulmonary disease. COPD J. Chronic Obstr. Pulm. Dis..

[3] Kent B.D., Mitchell P.D., McNicholas W.T. (2011). Hypoxemia in patients with COPD: Cause, effects, and disease progression. Int. J. Chronic Obstr. Pulm. Dis..

[4] Ussetti P., Rodriguez-Roisin R. (1991). Gas exchange during exercise in mild chronic obstructive pulmonary disease. Am. Rev. Respir. Dis..

[5] Dantzker D.R., D’Alonzo G.E. (1986). The effect of exercise on pulmonary gas exchange in patients with severe chronic obstructive pulmonary disease. Am. Rev. Respir. Dis..

[6] O’Donnell D.E., D’Arsigny C., Fitzpatrick M., Webb K.A. (2002). Exercise hypercapnia in advanced chronic obstructive pulmonary disease: The role of lung hyperinflation. Am. J. Respir. Crit. Care Med..

[7] Knower M.T., Dunagan D.P., Adair N.E., Chin R. (2001). Baseline oxygen saturation predicts exercise desaturation below prescription threshold in patients with chronic obstructive pulmonary disease. Arch. Intern. Med..

[8] Cutaia M., Brehm R., Cohen M. (2011). The relationship of the BODE index to oxygen saturation during daily activities in patients with chronic obstructive pulmonary disease. Lung.

[9] Zafar M.A., Tsuang W., Lach L., Eschenbacher W., Panos R.J. (2013). Dynamic hyperinflation correlates with exertional oxygen desaturation in patients with chronic obstructive pulmonary disease. Lung.

[10] Minh V.-D., Lee H.M., Dolan G.F., Light R.W., Bell J., Vasquez P. (1979). Hypoxemia during exercise in patients with chronic obstructive pulmonary disease. Am. Rev. Respir. Dis..

[11] Nowiński A., Kamiński D., Kram M., Korzybski D., Stokłosa A., Górecka D. (2013). Impact of mild anaemia on dyspnoea during exertion and exercise tolerance in patients with acute exacerbation of chronic obstructive pulmonary disease. Adv. Respir. Med..

[12] Moreira M.Â.F., Medeiros G.A.D., Boeno F.P., Sanches P.R.S., Silva D.P.D., Müller A.F. (2014). Oxygen desaturation during the six-minute walk test in COPD patients. J. Bras. Pneumol..

[13] Kim C., Seo J.B., Lee S.Y., Lee J.S., Huh J.W., Lee J.H., Ra S.W., Lee J.-H., Kim E.-K., Kim T.-H. (2013). Exertional desaturation as a predictor of rapid lung function decline in COPD. Respiration.

[14] Couillard A., Muir J.F., Veale D. (2010). COPD recent findings: Impact on clinical practice. COPD J. Chronic Obstr. Pulm. Dis..

[15] Tojo N., Ichioka M., Chida M., Miyazato I., Yoshizawa Y., Miyasaka N. (2005). Pulmonary exercise testing predicts prognosis in patients with chronic obstructive pulmonary disease. Intern. Med..

[16] Casanova C., Cote C., Marin J.M., Pinto-Plata V., de Torres J.P., Aguirre-Jaíme A., Vassaux C., Celli B.R. (2008). Distance and oxygen desaturation during the 6-min walk test as predictors of long-term mortality in patients with COPD. Chest.

[17] Stoller J.K., Panos R.J., Krachman S., Doherty D.E., Make B., Group L-tOTTR (2010). Oxygen therapy for patients with COPD: Current evidence and the long-term oxygen treatment trial. Chest.

[18] Group L-TOTTR (2016). A randomized trial of long-term oxygen for COPD with moderate desaturation. N. Engl. J. Med..

[19] Ameer F., Carson K.V., Usmani Z.A., Smith B.J. (2014). Ambulatory oxygen for people with chronic obstructive pulmonary disease who are not hypoxaemic at rest. Cochrane Database Syst. Rev..

[20] GROUP* NOTT (1980). Continuous or nocturnal oxygen therapy in hypoxemic chronic obstructive lung disease: A clinical trial. Ann. Intern. Med..

[21] Stuart H., Harris S., Tjh C., Dornhorst A.C., Cotes J.E., Flenley D.C., Howard P., Oldham P.D. (1981). Long term domiciliary oxygen therapy in chronic hypoxic cor pulmonale complicating chronic bronchitis and emphysema: Report of the Medical Research Council Working Party. Lancet.

[22] The Global Initiative for Chronic Obstructive Lung Disease: GOLD. http://www.goldcopd.com.

[23] Celli B., Cote C., Marín J., Casanova C., Montes de Oca Mendez R. (2004). The body mass index, airflow obstruction, dyspnea, exercise performance (BODE) index as a predictor of mortality in chronic obstructive pulmonary disease. N. Engl. J. Med..

[24] Poulain M., Durand F., Palomba B., Ceugniet F., Desplan J., Varray A. (2003). 6-minute walk testing is more sensitive than maximal incremental cycle testing for detecting oxygen desaturation in patients with COPD. Chest.

[25] Roberts M.M., Cho J.G., Sandoz J.S., Wheatley J.R. (2015). Oxygen desaturation and adverse events during 6-min walk testing in patients with COPD. Respirology.

[26] Pinto-Plata V., Cote C., Cabral H., Taylor J., Celli B. (2004). The 6-min walk distance: Change over time and value as a predictor of survival in severe COPD. Eur. Respir. J..

[27] Gould G., Redpath A., Ryan M., Warren P., Best J., Flenley D., MacNee W. (1991). Lung CT density correlates with measurements of airflow limitation and the diffusing capacity. Eur. Respir. J..

[28] Gould G.A., Redpath A.T., Ryan M., Warren P.M., Best J.J., Flenley D.C., MacNee W. (1996). Comparison of computed density and microscopic morphometry in pulmonary emphysema. Am. J. Respir. Crit. Care Med..

[29] Sue D.Y., Oren A., Hansen J.E., Wasserman K. (1987). Diffusing capacity for carbon monoxide as a predictor of gas exchange during exercise. N. Engl. J. Med..

[30] Hadeli K.O., Siegel E.M., Sherrill D.L., Beck K.C., Enright P.L. (2001). Predictors of oxygen desaturation during submaximal exercise in 8,000 patients. Chest.

[31] Soguel Schenkel N., Burdet L., de Muralt B., Fitting J.W. (1996). Oxygen saturation during daily activities in chronic obstructive pulmonary disease. Eur. Respir. J..

[32] Waatevik M., Johannessen A., Real F.G., Aanerud M. (2016). Oxygen desaturation in 6-min walk test is a risk factor for adverse outcomes in COPD. Eur. Respir. J..

[33] Fiore C., Lee A., McDonald C., Hill C.J., Holland A.E. (2011). Should oxyhaemoglobin saturation be monitored continuously during the 6-minute walk test?. Chronic Respir. Dis..

[34] Enright P.L. (2016). Oxygen desaturation during a 6-min walk identifies a COPD phenotype with an increased risk of morbidity and mortality. Eur. Respir. J..

[35] Liu S.-F., Wang C.-C., Chin C.-H., Chen Y.-C., Lin M.-C. (2011). High value of combined serum C-reactive protein and BODE score for mortality prediction in patients with stable COPD. Arch. Bronconeumol..

